# 14‐3‐3ζ binds to and stabilizes phospho‐beclin 1^S295^ and induces autophagy in hepatocellular carcinoma cells

**DOI:** 10.1111/jcmm.14806

**Published:** 2019-11-11

**Authors:** Yufu Tang, Yibing Zhang, Shupeng Liu, Zhongyi Sun, Chunhui Wang, Longfei Li, Wenping Zhou, Shuqun Cheng

**Affiliations:** ^1^ Department of Hepatobiliary Surgery The General Hospital of Northern Theater Command Shenyang China; ^2^ Department of Medical Affairs The General Hospital of Northern Theater Command Shenyang China; ^3^ Department of Gynecology and Obstetrics Tenth People’s Hospital Tongji University Shanghai China; ^4^ Department of Liver Surgery Eastern Hepatobiliary Surgery Hospital The Second Military Medical University Shanghai China

**Keywords:** 14‐3‐3ζ, autophagy, beclin 1, chemotherapy‐resistant, hepatocellular carcinoma, portal vein tumour thrombosis

## Abstract

Data from The Cancer Genome Atlas (TCGA) indicate that the expression levels of 14‐3‐3ζ and beclin 1 (a key molecule involved in cellular autophagy) are up‐regulated and positively correlated with each other (*R* = .5, *P* < .05) in HCC tissues. Chemoresistance developed in hepatoma cancer cells is associated with autophagy initiation. This study aimed to explore 14‐3‐3ζ’s role in regulating autophagy in HCC cells, with a focus on beclin 1. The co‐localization of 14‐3‐3ζ and beclin 1 was detectable in primary HCC tissues. To simulate in vivo tumour microenvironment (hypoxia), CSQT‐2 and HCC‐LM3 cells were exposed to 2% oxygen for 24 hours. The protein levels of 14‐3‐3ζ and phospho‐beclin 1^S295^ peaked at 12 hours following hypoxia. Meanwhile, the strongest autophagy flux occurred: LC3II was increased, and p62 was decreased significantly. By sequencing the coding area of *BECN 1* gene of CSQT‐2 and HCC‐LM3 cells, we found that the predicted translational products of *BECN 1* gene contained RLPS^295^VP (R, arginine; L, leucine; P, proline; S, serine; V, valine), a classic 14‐3‐3ζ binding motif. CO‐IP results confirmed that 14‐3‐3ζ bound to beclin 1, and this connection was markedly weakened when S^295^ was mutated into A^295^ (alanine). Further, 14‐3‐3ζ overexpression prevented phospho‐beclin 1^S295^ from degradation and enhanced its binding to VPS34, whilst its knockdown accelerated the degradation. Additionally, 14‐3‐3ζ enhanced the chemoresistance of HCC cells to cis‐diammined dichloridoplatium by activating autophagy. Our work reveals that 14‐3‐3ζ binds to and stabilizes phospho‐beclin 1^S295^ and induces autophagy in HCC cells to resist chemotherapy.

## 1 INTRODUCTION

Primary liver cancer comprises an unfavourable prognosis and is the second major cause of cancer‐associated death around the world.^1^ Hepatocellular carcinoma (HCC) that arises from the malignant transformation of hepatocytes is the major type of primary liver cancer.^2^ Portal vein tumour thrombosis (PVTT) is a severe complication that links to poor survival of patients with HCC.^3^ 14‐3‐3ζ (gene symbol *YWHAZ*) belongs to the highly conserved 14‐3‐3 protein family.^4^ By comparing to non‐tumour specimens, 14‐3‐3ζ expression was higher in tumour tissues derived from patiesnts with varied cancers, such as breast cancer and lung cancer.^5^, ^6^ Our group also found an up‐regulation in 14‐3‐3ζ in HCC tumour tissues, and this elevation might be associated with PVTT formation.^7^, ^8^ On the basis of our prior work, we continue exploring the role of 14‐3‐3ζ in HCC progress, with an emphasis on autophagy.

Autophagy is a catabolic mechanism by which the dysfunctional cytoplasmic components are degraded and recycled.^9^ It occurs to sustain the body homeostatic functions under basal conditions and displays both detrimental and protective capabilities upon the setting of pathological states.^10^ Low oxygen concentration (hypoxia) is commonly observed in the microenvironment of many solid malignancies.^11^ Cancer cells often activate pro‐survival autophagy to resist hypoxia‐induced death.^12^, ^13^, ^14^ We prior found that the expression level of hypoxia‐inducible factor 1α (HIF‐1α), a central hypoxia‐induced transcription factor, was higher in PVTT^(+)^ HCC tumour samples than that in PVTT^(−)^ tumour samples.^8^ In addition, we also demonstrated that 14‐3‐3ζ expression was increased in HCC cells exposed to hypoxia, and 14‐3‐3ζ itself could stabilize HIF‐1α, thereby promoting the growth and lung metastasis of HCC cells.^15^ Considering the important role of HIF‐1α in hypoxia‐induced autophagy,^16^ we propose that 14‐3‐3ζ affects HCC progress and PVTT formation by regulating autophagy.

Autophagy is precisely orchestrated by multiple autophagy‐related proteins (ATGs).^17^ A study from Zhao et al^18^ showed that ATG7 mRNA expression was up‐regulated after 14‐3‐3ζ overexpression and down‐regulated when 14‐3‐3ζ was knocked down in SMMC‐7721 cells. Weerasekara and coworkers proved that, under hypoxia, the phosphorylation of Atg9A at S761 (S, serine) was enhanced by AMPK, which enabled its binding to 14‐3‐3ζ, thereby promoting Atg9A recruitment to LC3‐positive autophagosomes.^19^ These two previous studies suggest a regulatory effect of 14‐3‐3ζ on autophagy‐associated molecules. It is worth noting that, once activated, autophagy proceeds through four sequential steps: initiation and nucleation of phagophore, autophagosome expansion, autolysosome maturation and autophagy execution.^10^, ^17^ Beclin 1 (gene symbol *BECN1*) that forms a complex with phosphatidylinositol‐3‐kinase (PI3K) is a vital molecule for triggering the very first step of autophagy.^10^, ^17^ To explore whether 14‐3‐3ζ affects autophagy by interacting beclin 1 will provide novel sights into 14‐3‐3ζ‐mediated autophagy.

As a phosphopeptide‐binding molecule, 14‐3‐3ζ primarily binds to proteins containing RXXpSXP or RXXXpSXP motifs (R, arginine; S or pS, serine or phospho‐serine; P, proline; X, any amino acid).^20^, ^21^ Interestingly, by analysing the amino acid sequence of *homo sapiens* beclin 1 (NM_003766), we noted that beclin 1 also contains RLPS^295^VP motif (R, arginine; L, leucine; P, proline; S, serine; V, valine). The phosphorylation of S^295^ in beclin 1 may create a 14‐3‐3ζ docking site. An earlier study from Song et al^22^ showed that the beclin 1 S^295^ can be phosphorylated. We thus assume that 14‐3‐3ζ can regulate autophagy by directly interacting with beclin 1.

The above‐mentioned hypothesis was tested in CSQT‐2 cells (established from PVTT tissue)^23^ and HCC‐LM3 cells^24^, two cell lines with high metastatic potential. We found that 14‐3‐3ζ indeed bound to beclin 1 by docking to RLPpS^295^VP motif and induced autophagy in these cells. 14‐3‐3ζ also prevented the protein degradation of phosphorylated beclin 1 in HCC cells exposed to transcriptional inhibitor.

## 2 MATERIALS AND METHODS

### 2.1 Database

The expression levels of *YWHAZ* and *BECN1* gene and their expression correlation were analysed with Gene Expression Profiling Interactive Analysis (GEPIA; http://gepia.cancer-pku.cn/; using data from The Cancer Genome Atlas [TCGA]). In short, the transcripts per million (TPM) of these two genes were detected in HCC tumours and non‐tumours. Further, their correlation coefficient in HCC samples was determined with Spearman's analysis. Positive *R* value indicated positive correlation. A *P* value <.05 was considered significant.

### 2.2 Cell culture and treatment

CSQT‐2 cells^23^ were stored in our laboratory and used as in vitro model for PVTT. HCC‐LM3 cells were kindly provided by Prof. Weizhong Wu (ZhongShan Hospital, Fu Dan University, Shanghai, China). Cells were maintained in DMEM (Gibco) containing 10% foetal bovine serum (Gibco) in a humidified incubator containing 5% CO_2_ at 37°C. For hypoxia, cells were cultured in 2% oxygen.

### 2.3 Reverse transcription‐polymerase chain reaction (RT‐PCR)

The encoding fragments of *BECN1* gene were amplified from CSQT‐2 cells and HCC‐LM3 cells by using a pair of primers: 5' cacaagcttatggaagggtctaagacgtc3' (underline, Hind III site); 5' cgcggatcctcatttgttataaaattgtgag 3' (underline, BamH I site) (whole fragment = 1371 bp; CDS = 1353 bp). The fragment size was confirmed via 1% agar electrophoresis (20 minutes). After purification, the fragments were sequenced in Sangon.

### 2.4 Eukaryotic vector

The c‐Flag pcDNA3 vector was obtained from Addgene (Addgene). The fragment encoding wild‐type beclin 1^S295^ (identical to NM_003766) was inserted into c‐Flag pcDNA3 vector between Hind III and BamH I sites. The beclin 1^S295A^ mutant was generated by specific primers that replaced AGT (a triplet codon of serine) with GCA (a triplet codon of alanine). The beclin 1^S295A^ was also constructed into c‐Flag pcDNA3 vector. Plasmid transfection into CSQT‐2 cells was mediated by Lipofectamine 2000 (Invitrogen) according to the manufactory's protocols.

### 2.5 Lentivirus vector

Tet‐pLKO‐puro (Addgene) and Psico‐GFP (Addgene) lentiviral vector systems were used to mediate the RNA interference (RNAi) of *YWHAZ* (14‐3‐3ζ) and BECN1 (beclin 1), respectively. Plv‐EF1a‐IRES‐neo lentiviral vector system (Addgene) was utilized to mediate the overexpression of 14‐3‐3ζ. Short hairpin sequences (shRNA) were as follows: *YWHAZ*‐(F)‐5'ccggcccaaaggagattactaccgttttcaagagaaacggtagtaatctcctttttttt 3', *YWHAZ*‐(R)‐5'aattaaaaaaaaggagattactaccgtttctcttgaaaacggtagtaatctcctttttttt3';

*BECN1*‐(F)‐5'tggacaacaagtttgaccatgcttcaagagagcatggtcaaacttgttgtccttttttc3',

*BECN1*‐(R)‐5'tcgagaaaaaaggacaacaagtttgaccatgctctcttgaagcatggtcaaacttgttgtcca3'. Lentiviral particles were packaged in HEK 293T cells, namely n.c. oe‐LV, 14‐3‐3ζ oe‐LV, n.c. sh‐LV, 14‐3‐3ζ sh‐LV and beclin 1 sh‐LV (n.c., negative control; oe, overexpression). Lentiviral particles of MOI = 20 were used to infect HCC cancer cells.

### 2.6 Quantitative real‐time polymerase chain reaction (qRT‐PCR)

In short, RNAs were isolated and processed into cDNAs a transcriptor first strand synthesis Super M‐MLV kit (BioTeke Bio). A pair of qRT‐PCR primers for *YWHAZ* was designed: *YWHAZ* forward, 5' gccattgctgaacttgata 3'; *YWHAZ* reverse: 5' gcttcgtctccttgggtat 3'. The mRNA expression levels were calculated based on 2^‐ΔΔct^ by using SYBR Premix Ex TaqTM (TaKaRa).

### 2.7 Co‐immunoprecipitation (co‐IP) and Western blot

For co‐IP, cell proteins were first extracted from cancer cells, and then, protein (200 μg) was incubated with 1 μL anti‐beclin 1 antibody or ant‐flag antibody overnight. Then, these samples were incubated with 60 μL Protein A Agarose at 2°C for 2 hours. After centrifugation, the mixture was rinsed with 1 × PBS and then resuspended in 60 μL loading buffer (5×). After being boiled for 5 minutes, the sample was subjected for Western blot.

For Western blot, equal protein sample was separated on a 10% SDS‐PAGE and transferred onto PVDF membrane. After blocking via skim milk, the PVDF membrane was incubated with one of the following primary antibodies overnight: anti‐14‐3‐3ζ antibody (1:1000; CST, Danvers), anti‐beclin 1 (1:1000; CST,), anti‐phospho‐beclin 1^S295^ (1:500; Abcam), anti‐LC3 (1:1000; ProteinTech), anti‐p62 (1:1000; CST) or anti‐vacuolar protein sorting 34 (VPS34; 1:1000; Abclonal). Thereafter, the membrane was incubated with goat anti‐rabbit secondary antibody (1:5000; Abcam). β‐Actin was the reference. The intensities of LC3‐II and LC3‐I were compared to that of β‐actin, and the ratio was calculated as the following equation: (LC3‐II/β‐actin)/(LC3‐I/β‐actin). For some experiments, cells were pretreated with 20 mg/mL actinomycin D (MedChemExpress).

### 2.8 Patients

Tissue samples were obtained from a patient diagnosed with HCC/PVTT before neoadjuvant therapy. A written informed consent was provided by this patient. The research was carried out according to the World Medical Association Declaration of Helsinki.

### 2.9 Immunofluorescence

Cells were fixed in 4% paraformaldehyde and permeabilized in tritonX‐100 (0.1%) at room temperature for half an hour. Primary HCC tissue samples were cut into 5‐μm sections and deparaffinized. After dehydration, tissue slices were incubated in boiled antigen retrieval buffer and soaked in 1× PBS for a total of 15 minutes. Both cells and tissue slices were incubated in goat serum at room temperature and then incubated with one of the following antibodies: LC3 (1:200, CST), anti‐14‐3‐3ζ (1:100 for cells; 1:50 for tissue slices; Sangon Biotech) and anti‐beclin 1 (1:200 for cells; 1:50 for tissue slices; ProteinTech) overnight. Then, cells were treated with FITC‐ or Cy3‐labelled secondary antibody. Cell nuclei were counterstained with DAPI. 14‐3‐3ζ‐ and beclin 1‐positive cells were counted and shown as a percentage.

### 2.10 Flow cytometry assay

Experiment 1: Cells were infected with 14‐3‐3ζ oe‐LV or n.c. oe‐LV. Forty‐eight hours later, cells were treated with 100 μmol/L cis‐diammined dichloridoplatium (CDDP; Meilun Biotech), 10 mmol/L 3‐MA (MedChemExpress) and 30 μmol/L chloroquine (MedChemExpress) for additional 24 hours.

Experiment 2: Cells were co‐infected with 14‐3‐3ζ oe‐LV and beclin 1 sh‐LV. Forty‐eight hours later, cells were treated with 100 μmol/L CDDP for 24 hours.

To determine the apoptotic ratios, cells were stained with Annexin V‐Light 650/PI (Wanleibio) and analysed on a flow cytometry.

### 2.11 Statistical analysis

All data were presented in mean plus standard deviation (SD) and analysed with SPSS version 20.0 (IBM). Comparison between two groups was performed with unpaired Student's *t* test (two tailed). Comparison over multiple groups was performed with one‐way ANOVA followed by Bonferroni's multiple comparison test. A *P* value <.05 was considered significant.

## 3 RESULTS

### 3.1 Positive expression correlation of *YWHAZ* and *BCEN1* gene in HCC

**Figure 1:**
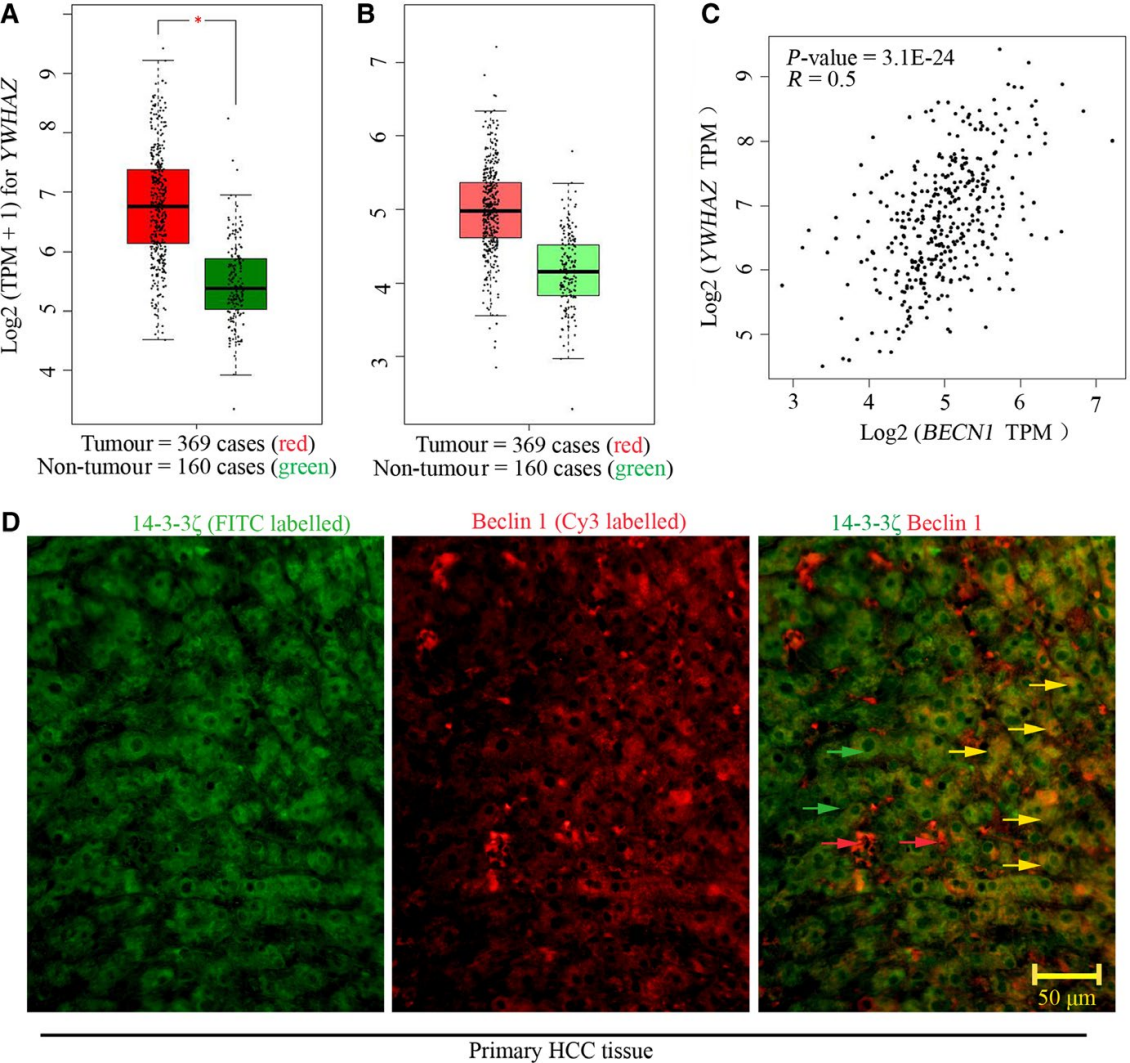
Positive expression correlation of *YWHAZ* and *BCEN1* gene in HCC. GEPIA public database was used to analyse the transcripts per million of (A) *YWHAZ* and (B) *BCEN1* gene in samples derived from patients with HCC. (C) The expression correlation of these two genes was analysed with Spearman. (D) Immunofluorescence was performed to probe 14‐3‐3ζ and beclin 1 expression in primary HCC tissue. Green arrows indicated 14‐3‐3ζ, whereas red arrows indicated beclin 1. Yellow arrows indicated the colocalization of 14‐3‐3ζand beclin 1. Positive *R* value indicated positive correlation. A *P value <.05 was considered significant. *BCEN1*, beclin 1; GEPIA, Gene Expression Profiling Interactive Analysis

By analysing the TPM of *YWHAZ* and *BCEN1* gene via GEPIA public database, we found that their expression levels were concomitantly higher in HCC tumours (n = 369) than that in non‐tumours (n = 160; Figure 1A,B). Further, Spearman's results indicated that *YWHAZ* expression was positively correlated with *BCEN1* expression (*P* value <.05; *R* = .5; Figure 1C)*.* In addition, immunofluorescence was performed to determine 14‐3‐3ζ (green) and beclin 1 (red) expression. 14‐3‐3ζ and beclin 1 were detectable in the primary HCC tissue, and their co‐localization could be observed (Figure 1D).

### 3.2 Hypoxia induces 14‐3‐3ζ expression and promotes autophagy in HCC‐LM3 and CSQT‐2 cells

**Figure 2:**
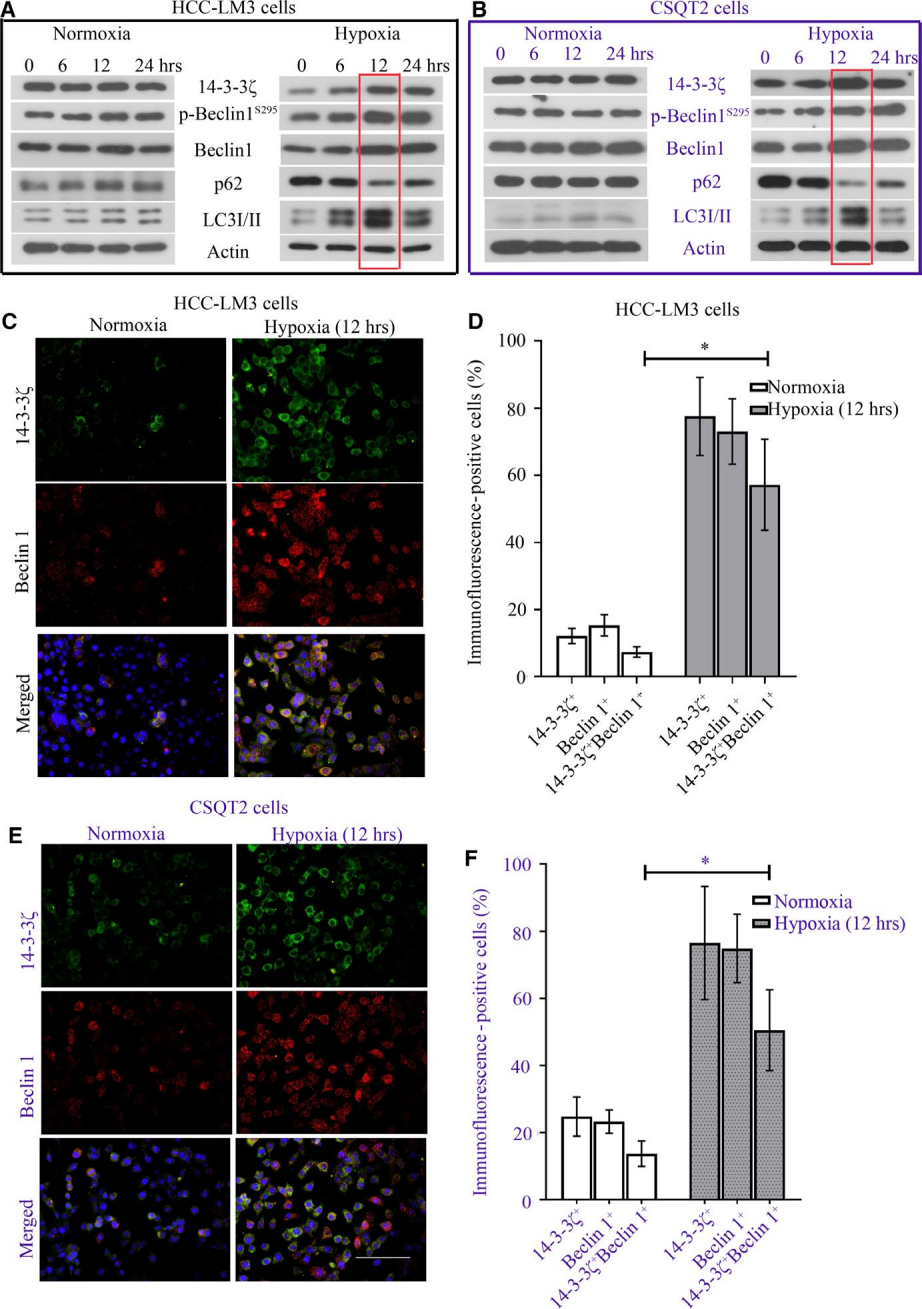
Hypoxia induces 14‐3‐3ζ expression and promotes autophagy in HCC‐LM3 and CSQT‐2 cells. HCC‐LM3 and CSQT‐2 cells were cultured in 2% oxygen (Hypoxia) or 95% air (Normoxia) for 6, 12 or 24 h. (A,B) The protein levels of 14‐3‐3ζ and beclin 1, phospho‐beclin 1^S295^, LC3 and p62 were determined with Western blot analysis. Cancer cells were cultured under hypoxia for 12 h, and (C,E) immunofluorescence was performed to probe 14‐3‐3ζ and beclin 1 expression. Green fluorescence marked 14‐3‐3ζ, whereas red fluorescence marked beclin 1. (D,F) 14‐3‐3ζ‐ and beclin 1‐positive cells were counted and shown as a percentage. A *P value <.05 was considered significant

HCC‐LM3 and CSQT‐2 cells were cultured in hypoxic condition for 6, 12 or 24 hours. The protein levels of 14‐3‐3ζ and autophagy‐associated markers, beclin 1, phospho‐beclin 1^S295^, LC3 and p62, were determined with Western blot analysis. As indicated in Figure 2, we found that the protein levels of these molecules were hardly changed in cells under normoxia. Hypoxia up‐regulated 14‐3‐3ζ, beclin 1, phospho‐beclin 1^S295^ and LC3II levels (Figure S1) and down‐regulated p62 expression in HCC‐LM3 and CSQT‐2 cells (Figure 2A,B). The most significant changes in these proteins were observed at 12 hours after hypoxia (Figure 2A,B). Thus, for following experiments related to hypoxia, cancer cells were cultured for 12 hours. Immunofluorescence double staining using anti‐14‐3‐3ζ and anti‐beclin 1 antibodies indicated that 14‐3‐3ζ and beclin 1 expression levels were up‐regulated in response to a 12‐hour hypoxia (Figure 2C,D). They preferred to co‐localizing with each other under hypoxia (Figure 2C‐F). These data demonstrate that hypoxia‐induced autophagy is accompanied with 14‐3‐3ζ elevation.

### 3.3 14‐3‐3ζ promotes autophagy in HCC‐LM3 and CSQT‐2 cells

**Figure 3:**
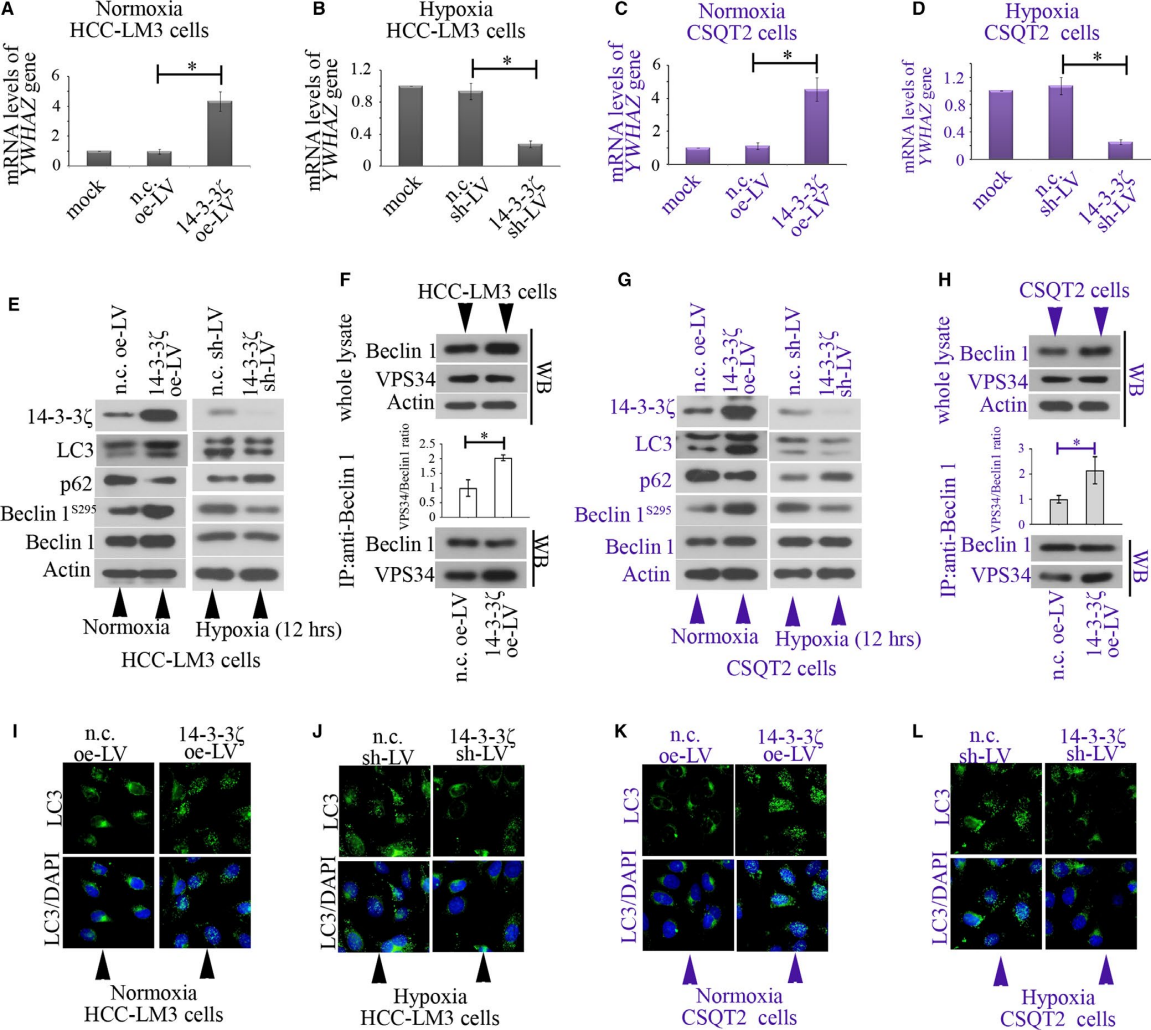
14‐3‐3ζ induces autophagy in HCC‐LM3 and CSQT‐2 cells. (A) HCC‐LM3 and (C) CSQT‐2 cells were infected with n.c. oe‐LV or 14‐3‐3ζ oe‐LV for 72 h, and the mRNA levels of *YWHAZ* gene were determined with qRT‐PCR and normalized to β‐actin. For knockdown detection, cells were first infected with n.c. sh‐LV or 14‐3‐3ζ sh‐LV for 72 h and then incubated under hypoxia for additional 12 h. The expression mRNA levels of *YWHAZ* gene in (B) HCC‐LM3 and (D) CSQT‐2 cells were determined with qRT‐PCR and normalized to β‐actin. (E‐H) Western blot was performed to determine the protein levels of 14‐3‐3ζ, p62, beclin 1 and phospho‐beclin 1^S295^. (F,H) Co‐IP with anti‐beclin 1 for IP, and anti‐beclin 1 and anti‐VPS34 for IB was performed. (I‐L) Immunofluorescence was performed to analyse the formation of LC3 puncta. Data were shown in mean with SD (N = 3). A *P value <.05 was considered significant. nc, negative control; LV, lentivirus; oe, overexpression; shRNA, short hairpin RNA

HCC‐LM3 and CSQT‐2 cells were infected with n.c. oe‐LV or 14‐3‐3ζ oe‐LV for 72 hours, and the expression of mRNA and protein levels of *YWHAZ* were determined with qRT‐PCR and Western blot (Figure 3A,C,E,G). A significant up‐regulation of *YWHAZ* at both transcriptional and translational levels confirmed the efficiency of 14‐3‐3ζ oe‐LV. For knockdown detection, cells were first infected with n.c. sh‐LV or 14‐3‐3ζ sh‐LV for 72 hours and then incubated under hypoxia for additional 12 hours. 14‐3‐3ζ sh‐LV could effectively knock 14‐3‐3ζ down in these two cell lines (Figure 3B,D,E,G).

We found that 14‐3‐3ζ overexpression could induce autophagy in HCC‐LM3 and CSQT‐2 cells: LC3I to LC3II conversion was enhanced, and LC3II puncta were more (Figure 3E,G,I,K); the p62 levels were decreased (Figure 3E,G). The total protein expression of beclin 1 was slightly increased, whereas phospho‐beclin 1^S295^ was significantly up‐regulated in cells overexpressing 14‐3‐3ζ (Figure 3E,G). The overexpression of 14‐3‐3ζ also up‐regulated the mRNA expression of *BECN1* gene in cancer cells (Figure S2). Moreover, 14‐3‐3ζ overexpression did not alter the expression of VPS34 in HCC cells, but augmented its binding to beclin‐1 (Figure 3F,H).

Under hypoxia, 14‐3‐3ζ sh‐LV decreased phospho‐beclin 1^S295^ level, without affecting the total beclin 1 level. Moreover, hypoxia‐induced autophagy was suppressed when 14‐3‐3ζ was silenced (Figure 3 E,G). Less LC3II puncta were observed in 14‐3‐3ζ‐silenced cells under hypoxia (Figure 3J,L). These findings illustrate that 14‐3‐3ζ enhances autophagy in HCC cancer cells.

### 3.4 14‐3‐3ζ binds to phospho‐beclin 1^S295^ in HCC‐LM3 and CSQT‐2 cells

**Figure 4:**
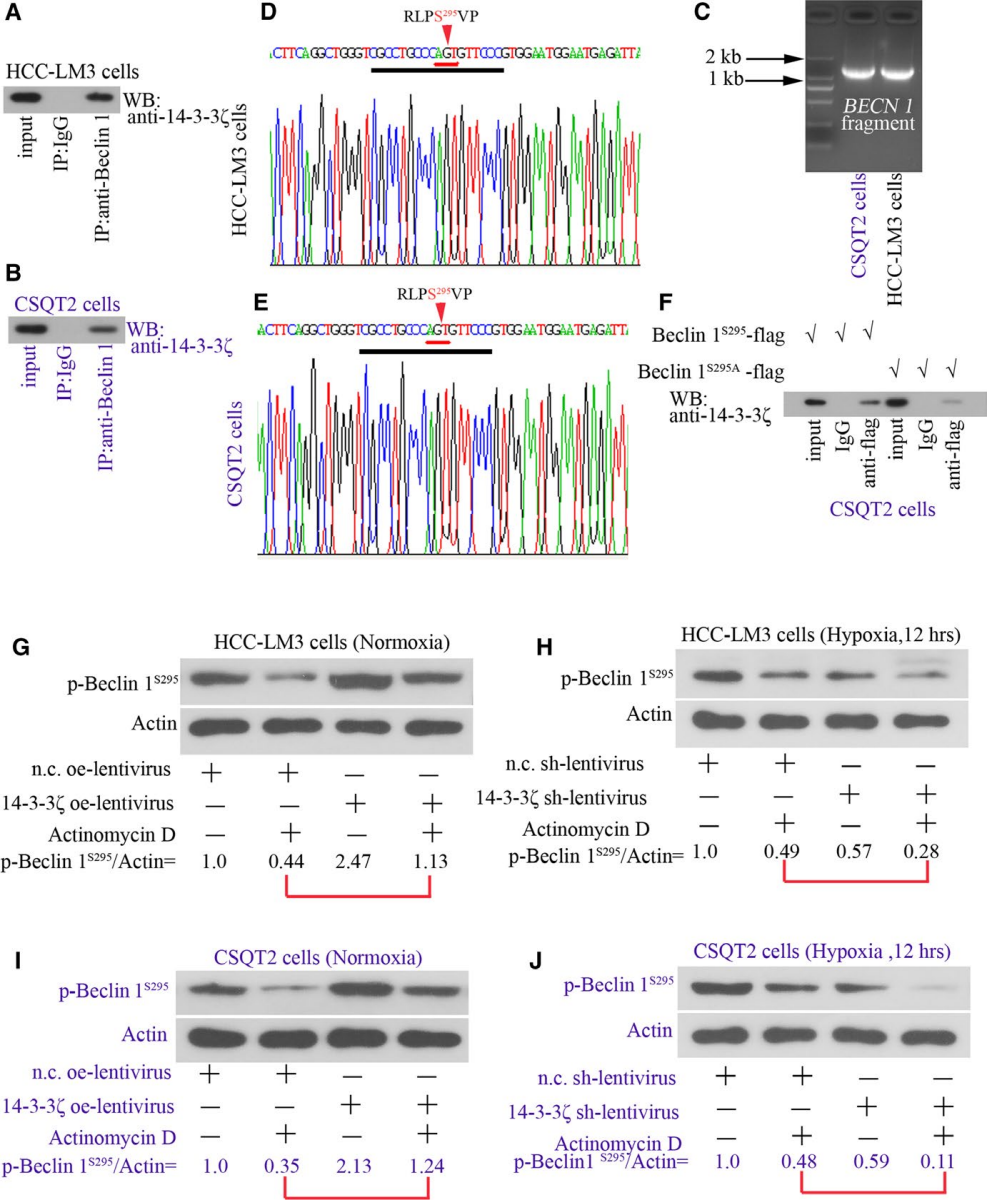
14‐3‐3ζ binds to phospho‐beclin 1^S295^ in HCC‐LM3 and CSQT‐2 cells. Co‐IP was conducted in both (A) HCC‐LM3 and (B) CSQT‐2 cells. (C) Fragments (1371 bp) containing *BECN 1* gene CDS were amplified from these cells and analysed with agar electrophoresis. The sequence information closely to RLPS^295^VP (R, arginine; L, leucine; P, proline; S, serine; V, valine) was shown in D,E. (F) Flag‐labelled beclin 1^S295^ and beclin 1^S295A^ were transfected into CSQT‐2 cells, and co‐IP with anti‐flag antibody for IP and anti‐14‐3‐3ζ antibody for IB was performed. (G) HCC‐LM3 and (I) CSQT‐2 cells were infected with n.c. oe‐LV or 14‐3‐3ζ oe‐LV, and 72 h later, cells were treated with 20 mg/mL actinomycin D for additional 12 h. Protein levels of phospho‐beclin 1^S295^ were assessed with Western blot. In addition, (H) HCC‐LM3 and (J) CSQT‐2 cells were infected with n.c. sh‐LV or 14‐3‐3ζ sh‐LV, and 72 h later, cells were treated with 20 mg/mL actinomycin D for additional 12 h under hypoxia. Protein levels of phospho‐beclin 1^S295^ were also assessed with Western blot. β‐Actin was the reference. nc, negative control; LV, lentivirus; oe, overexpression; shRNA, short hairpin RNA

CO‐IP was conducted in both HCC‐LM3 and CSQT‐2 cells. A co‐localization of 14‐3‐3ζ and beclin 1 was observed in both HCC‐LM3 and CSQT‐2 cells (Figure 4A,B). The encoding fragments of *BECN1* gene were amplified from CSQT‐2 cells and HCC‐LM3 cells and analysed with agar electrophoresis (Figure 4C). The fragments with correct size were then sequenced. We found that the amplified fragments shared identical nucleic acid information with *BECN 1*‐NM_003766. The predicted polypeptides encoded by this fragment contained RLPS^295^VP, a potential motif for 14‐3‐3ζ binding (Figure 4D,E). Next, flag‐labelled beclin 1^S295^ and beclin 1^S295A^ were transfected into CSQT‐2 cells, and CO‐IP with anti‐flag antibody for IP and anti‐14‐3‐3ζ antibody for IB was performed. The results showed that phosphorylation of beclin 1 on S295 was responsible for 14‐3‐3ζ binding. These data identify beclin 1 as a novel protein that interacts with 14‐3‐3ζ.

Furthermore, HCC‐LM3 and CSQT‐2 cells were treated with transcriptional inhibitor, actinomycin D. The degradation of phospho‐beclin 1 was slower in cells infected with 14‐3‐3ζ oe‐LV than in cells infected with n.c. oe‐LV (Figure 4G,I). 14‐3‐3ζ oe‐LV infection was confirmed to promote autophagy in terms of enhanced LC3 II conversion (Figure S3).

Under hypoxia, phospho‐beclin 1 degraded much faster in 14‐3‐3ζ‐silenced cells (Figure 4H,J). These results confirm that 14‐3‐3ζ binds to and stabilizes beclin 1 protein.

### 3.5 14‐3‐3ζ contributes chemoresistance of HCC cells to CDDP by inducing autophagy

**Figure 5:**
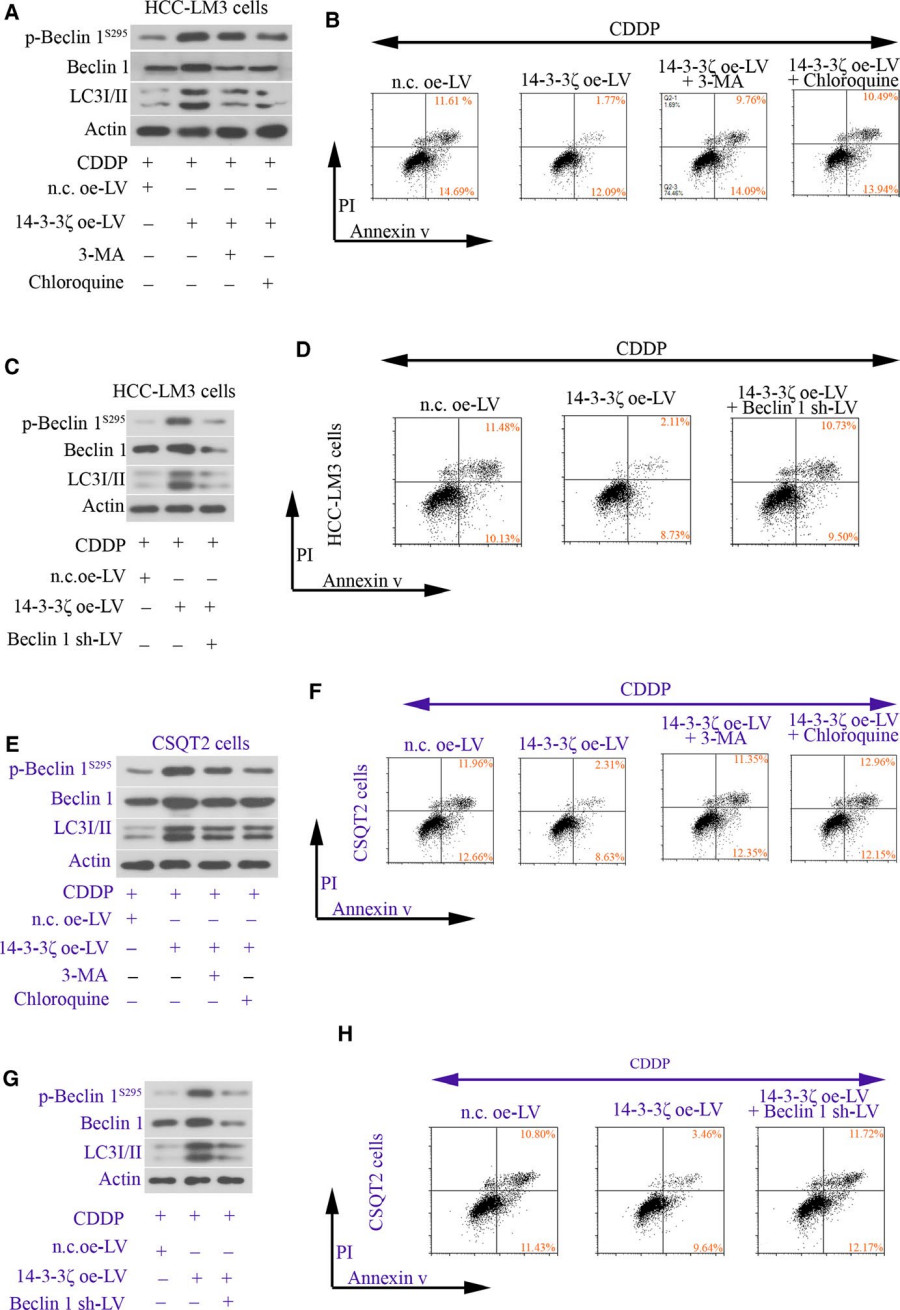
14‐3‐3ζ contributes chemoresistance of HCC cells to CDDP by inducing autophagy. HCC‐LM3 and CSQT2 cells were infected with n.c. oe‐LV, 14‐3‐3ζ oe‐LV or beclin 1 sh‐LV, and 48 h later, cells were treated with 100 μmol/L CDDP, 10 mmol/L 3‐MA or 30 μmol/L chloroquine for additional 24 h. (A, C, E,G) The protein levels of phospho‐beclin 1^S295^, beclin 1 and LC3 were determined with Western blot analysis. (B, D, F,H) Apoptotic cells were stained with Annexin V/PI and analysed via flow cytometry assay. nc, negative control; LV, lentivirus; oe, overexpression; shRNA, short hairpin RNA; CDDP, cis‐diammined dichloridoplatium

Cis‐diammined dichloridoplatium of 100 μmol/L was used to treat HCC‐LM3 and CSQT‐2 cells for 24 hours. Apoptotic cells were stained with Annexin V/PI and analysed via flow cytometry assay. By analysing the expression of autophagy‐associated molecules with Western blot, 3‐MA, chloroquine or beclin 1 sh‐LV could effectively reduce autophagy flux: LC3II level was significantly reduced (Figure 5A,C,E,G). Further, 14‐3‐3ζ overexpression inhibited CDDP‐induced apoptosis in these two cell line (Figure 5B,F). This effect was weakened when cells were co‐treated with 3‐MA or chloroquine (Figure 5B,F). Similar alterations were observed in cells co‐infected with 14‐3‐3ζ oe‐LV and/or beclin 1 sh‐LV (Figure 5D,H). These data confirm that 14‐3‐3ζ overexpression activates autophagy in HCC cells to induce their resistance to CDDP.

## 4 DISCUSSION

**Figure 6:**
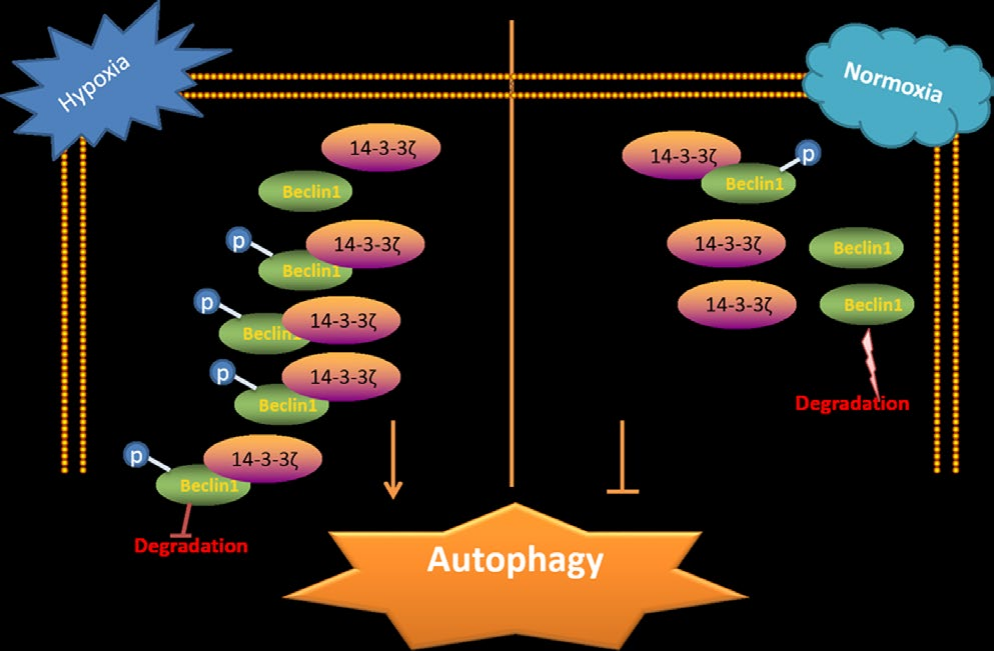
Summary of major findings. In hepatocellular carcinoma (HCC) tumour microenvironment (hypoxia), the levels of 14‐3‐3ζ and phospho‐beclin 1^S295^ are up‐regulated. 14‐3‐3ζ can bind to and prevent the phosphorylated beclin 1 from degradation in HCC cancer cells, thus promoting autophagy

Data from GEPIA revealed a positive correlation between *YWHAZ* and *BCEN1*. Furthermore, our in vitro results demonstrated that 14‐3‐3ζ bound to and stabilized phospho‐beclin 1^S295^ in HCC‐LM3 and CSQT2 cells (Figure 6) and induced autophagy in these cells to resist CDDP cytotoxicity. The RLPS^295^VP of beclin 1 was confirmed to provide the docking site for 14‐3‐3ζ.

During autophagy, beclin 1 consists a complex with PI3K to induce the initiation/nucleation of autophagosome. Sequentially, soluble LC3I converses to autophagosome‐associated LC3II. Finally, p62 shuttles the aggregates of intracellular proteins into autolysosome for degradation.^10^, ^17^ Herein, HCC‐LM3 and CSQT‐2 cells were exposed to hypoxia up to 24 hours to mimic the in vivo tumour microenvironment. The strongest autophagy flux was observed in these cells at 12 hours after hypoxia: LC3II was increased, and p62 was decreased markedly. Interestingly, we noted that 14‐3‐3ζ protein level in these cancer cells also peaked at 12 hours after hypoxia. This concurrent event suggests that 14‐3‐3ζ elevation may participate in hypoxia‐induced autophagy.

Beclin 1 contains several sites that can be phosphorylated by kinases. For instance, death‐associated protein kinase (DAPK) phosphorylates beclin 1 on T119,^25^ whereas AMPK phosphorylates beclin 1 on S90 and S93 in an ATG14‐dependent manner.^26^ Such phosphorylation on beclin 1 is important for autophagy initiation. Notably, hypoxia can phosphorylate beclin 1 at certain site as well—beclin 1 S30 was phosphorylated in U87 cells under hypoxia.^27^ In the present study, we first analysed the sequence information of *BECN1* gene in both HCC‐LM3 and CSQT‐2 cells and found that the encoding region of *BECN1* gene of the two cell lines were identical to *BECN1*‐NM_003766 uploaded on protein database of National Center for Biotechnology Information (NCBI). The sequence information implied that S295, but not S30, may provide a binding site for 14‐3‐3ζ. Thus, we additionally detected the protein level of phospho‐beclin 1^S295^ in HCC‐LM3 and CSQT‐2 cells under normoxic and hypoxic conditions. Our data revealed a novel phenomenon that hypoxic exposure can induce phosphorylation of beclin 1 on S295.

Considering that 14‐3‐3ζ may interact with phospho‐beclin 1^S295^, further experiments were performed to investigate whether 14‐3‐3ζ played a role in autophagy by regulating beclin 1. As both HCC‐LM3 and CSQT‐2 cell lines expressed wild‐type beclin 1, CO‐IP was first performed to determine the 14‐3‐3ζ‐beclin 1 binding in these cells. The corresponding data proved that 14‐3‐3ζ did bind to beclin 1. Next, flag‐labelled wild‐type beclin 1^S295^ and mutant‐type beclin 1^S295A^ plasmids were transfected into CSQT‐2 cells. Additional CO‐IP results demonstrate that phosphorylation of beclin 1 on S295 is responsible for 14‐3‐3ζ binding, identifying beclin 1 as a novel protein that interacts with 14‐3‐3ζ. Notably, mutation of S295 into A295 did not completely shut down the binding of 14‐3‐3ζ to beclin 1 (Figure 4). One explanation is that there is 14‐3‐3ζ binding area on beclin 1 other than RLPS^295^VP. Several previous studies have shown that proteins containing non‐classical binding domain for 14‐3‐3s can also be recognized by 14‐3‐3s.^28^, ^29^ The weak binding of 14‐3‐3ζ to beclin 1^A295^ may be attributed to the phosphorylation of other serine sites on beclin 1.

Interestingly, both the *BECN 1* mRNA and total protein expression levels were increased when 14‐3‐3ζ was overexpressed, implying that 14‐3‐3ζ promoted the transcription and translation of *BECN 1* gene in HCC cells. Of note, in SMMC‐7721 cells, 14‐3‐3ζ positively regulated the mRNA expression of *ATG7* gene.^18^ Such findings together with our current data suggest that 14‐3‐3ζ may act as a transcription activator. However, to our knowledge, 14‐3‐3ζ is not a transcription factor. Why the transcription of *BECN1* gene was initiated by 14‐3‐3ζ is unclear. We previously demonstrated that 14‐3‐3ζ was able to stabilize HIF‐1α, a well‐known transcription factor.^15^ Such results are supported by a study conducted in non–small‐cell lung cancer cells.^30^ 14‐3‐3ζ itself may not be a transcription factor, but it may affect other transcription factors, such as HIF‐1α, to indirectly regulate *BECN1* gene transcription. We additionally knocked down 14‐3‐3ζ in hypoxic cancer cells and found that, under hypoxia, 14‐3‐3ζ barely affected *BECN1* gene expression at translational level. These data suggest that once the hypoxic pathway is activated, 14‐3‐3ζ is not the predominant regulator of *BECN1* transcription or translation.

Moreover, we also analysed the protein levels of phospho‐beclin 1^S295^ in HCC cells overexpressing 14‐3‐3ζ. Notably, we found that 14‐3‐3ζ overexpression markedly promoted the phosphorylation of beclin 1 on S295. No kinase activity of 14‐3‐3ζ has been reported before. We then ask why 14‐3‐3ζ overexpression promotes beclin 1 phosphorylation. Previous studies report that 14‐3‐3s affect the stabilization of their binding partners. We therefore proposed that the observed elevation in phosphorylated beclin 1 was because 14‐3‐3ζ could prevent its degradation. The roles of 14‐3‐3s in regulating protein degradation are under debate. Whereas Wang et al^29^ demonstrated 14‐3‐3τ interacted with phospho‐E2F transcription factor 1 (E2F1) during DNA damage and inhibited E2F1 degradation, Mohammad et al^31^ proved that binding of 14‐3‐3ζ to phospho‐Bruton's tyrosine kinase (Btk) stimulated Btk degradation. Herein, in the presence of actinomycin D, the degradation of phospho‐beclin 1 was prevented in cells overexpressing 14‐3‐3ζ. Under hypoxia, phospho‐beclin 1 degraded faster in 14‐3‐3ζ‐silenced cells in contrast. To confirm that 14‐3‐3ζ indeed stabilizes phospho‐beclin 1 in HCC cells, treating cells with protease inhibitors, such as MG132 or bafimomycin, is urgently needed. Whether ubiquitin‐proteasome pathway, a key pathway that is involved in proteolysis, particulates in 14‐3‐3ζ‐mediated beclin 1 stability also requires further exploration.

VPS34 is the only class III PI3K in mammals that forms a stable complex with p150, which further associates with beclin‐1. The VPS34‐p150‐beclin‐1 complex serves as a binding partner for several proteins, such as Atg14, to promote autophagy.^32^, ^33^ Enhancing the interaction between VPS34 and beclin‐1 is capable of promoting autophagy.^34^, ^35^ Our data illustrated that 14‐3‐3ζ overexpression enhanced the binding of VPS34 and beclin‐1 and further triggered autophagy in HCC cells. These findings suggest that 14‐3‐3ζ can augment the formation of PI3K complex.

By analysing the protein levels of autophagy markers, we also found that, in addition to regulating beclin 1, 14‐3‐3ζ overexpression induced effective autophagic flux in HCC‐LM3 and CSQT‐2 cells—p62 degradation was enhanced and the number of LC3II puncta was increased. Moreover, hypoxia‐induced autophagy was suppressed in 14‐3‐3ζ‐silenced cells. Chemoresistance represents a major obstacle in clinical oncology. It is commonly considered that chemoresistance developed in cancer cells is associated with autophagy initiation.^36^ Suppression of 14‐3‐3ζ has been reported to promote chemosensitivity of several types of cancer cells, such as lung and breast cancer.^37^, ^38^ Choi and coworkers demonstrated that silencing of 14‐3‐3ζ enhanced the cytotoxicity of CDDP in many hepatoma cell lines.^39^ These previous literatures depict 14‐3‐3ζ as a contributor to the chemoresistance of cancer cells. However, whether autophagy is involved in 14‐3‐3ζ‐mediated chemoresistance is unknown. In agreement with earlier findings,^39^ we demonstrated that 14‐3‐3ζ overexpression promoted the resistance of HCC‐LM3 and CSQT‐2 cells to CDDP. Furthermore, 14‐3‐3ζ‐induced chemoresistance and LC3I to LC3II conversion were attenuated by autophagy inhibitors or by *BECN 1* silencing. Our data suggest that HCC cells with high 14‐3‐3ζ expression activate beclin 1‐mediated autophagy to resist cytotoxicity induced by chemotherapeutic drug.

In conclusion, we identify beclin 1 as a novel molecule that can be regulated by 14‐3‐3ζ. The RLPS^295^VP of beclin 1 provides the docking site for 14‐3‐3ζ. 14‐3‐3ζ can bind to stabilize phospho‐beclin 1^S295^ in HCC‐LM3 and CSQT2 cancer cells. 14‐3‐3ζ overexpression contributes to chemoresistance of HCC cells by activating autophagy.

## CONFLICT OF INTEREST

The authors confirm that there are no conflicts of interest.

## AUTHOR CONTRIBUTION

Yufu Tang designed and performed the research, analysed data and wrote the paper. Yibing Zhang, Shupeng Liu and Zhongyi Sun performed the research, analysed data and revised the paper. Chunhui Wang and Longfei Li analysed the data. Wenping Zhou and Shuqun Cheng designed the study and revised the paper. All authors approved the submitted final version.

## Data Availability

The data that support the findings of this study are openly available.

## References

[1] Sia D , Villanueva A , Friedman SL , Llovet JM . Liver cancer cell of origin, molecular class, and effects on patient prognosis. Gastroenterology. 2017;152:745‐761. 28043904 10.1053/j.gastro.2016.11.048 PMC12160040

[2] Calderaro J , Couchy G , Imbeaud S , et al. Histological subtypes of hepatocellular carcinoma are related to gene mutations and molecular tumour classification. J Hepatol. 2017;67:727‐738. 28532995 10.1016/j.jhep.2017.05.014

[3] Guo W , Xue J , Shi J , et al. Proteomics analysis of distinct portal vein tumor thrombi in hepatocellular carcinoma patients. J Proteome Res. 2010;9:4170‐4175. 20583822 10.1021/pr100412w

[4] Mhawech P . 14‐3‐3 proteins–an update. Cell Res. 2005;15:228‐236. 15857577 10.1038/sj.cr.7290291

[5] Xu J , Acharya S , Sahin O , et al. 14‐3‐3zeta turns TGF‐beta's function from tumor suppressor to metastasis promoter in breast cancer by contextual changes of Smad partners from p53 to Gli2. Cancer Cell. 2015;27:177‐192. 25670079 10.1016/j.ccell.2014.11.025 PMC4325275

[6] Li Z , Zhao J , Du Y , et al. Down‐regulation of 14‐3‐3zeta suppresses anchorage‐independent growth of lung cancer cells through anoikis activation. Proc Natl Acad Sci USA. 2008;105:162‐167. 18162532 10.1073/pnas.0710905105 PMC2224179

[7] Tang Y , Zhang Y , Wang C , et al. 14‐3‐3zeta binds to hepatitis B virus protein X and maintains its protein stability in hepatocellular carcinoma cells. Cancer Med. 2018;7:5543‐5553. 30358169 10.1002/cam4.1512 PMC6247021

[8] Tang Y , Liu S , Li N , et al. 14‐3‐3zeta promotes hepatocellular carcinoma venous metastasis by modulating hypoxia‐inducible factor‐1alpha. Oncotarget. 2016;7:15854‐15867. 26910835 10.18632/oncotarget.7493 PMC4941282

[9] Kundu M , Thompson CB . Autophagy: basic principles and relevance to disease. Annu Rev Pathol. 2008;3:427‐455. 18039129 10.1146/annurev.pathmechdis.2.010506.091842

[10] Vindis C . Autophagy: an emerging therapeutic target in vascular diseases. Br J Pharmacol. 2015;172:2167‐2178. 25537552 10.1111/bph.13052 PMC4403086

[11] Cairns RA , Papandreou I , Sutphin PD , Denko NC . Metabolic targeting of hypoxia and HIF1 in solid tumors can enhance cytotoxic chemotherapy. Proc Natl Acad Sci USA. 2007;104:9445‐9450. 17517659 10.1073/pnas.0611662104 PMC1890514

[12] Abdul Rahim SA , Dirkse A , Oudin A , et al. Regulation of hypoxia‐induced autophagy in glioblastoma involves ATG9A. Br J Cancer. 2017;117:813‐825. 28797031 10.1038/bjc.2017.263 PMC5590001

[13] Spowart JE , Townsend KN , Huwait H , et al. The autophagy protein LC3A correlates with hypoxia and is a prognostic marker of patient survival in clear cell ovarian cancer. J Pathol. 2012;228:437‐447. 22926683 10.1002/path.4090

[14] Gariboldi MB , Taiana E , Bonzi MC , et al. The BH3‐mimetic obatoclax reduces HIF‐1alpha levels and HIF‐1 transcriptional activity and sensitizes hypoxic colon adenocarcinoma cells to 5‐fluorouracil. Cancer Lett. 2015;364:156‐164. 25979228 10.1016/j.canlet.2015.05.008

[15] Tang Y , Lv P , Sun Z , Han L , Luo B , Zhou W . 14‐3‐3zeta up‐regulates hypoxia‐inducible factor‐1alpha in hepatocellular carcinoma via activation of PI3K/Akt/NF‐small ka, CyrillicB signal transduction pathway. Int J Clin Exp Pathol. 2015;8:15845‐15853. 26884855 PMC4730068

[16] Zhang H , Bosch‐Marce M , Shimoda LA , et al. Mitochondrial autophagy is an HIF‐1‐dependent adaptive metabolic response to hypoxia. J Biol Chem. 2008;283:10892‐10903. 18281291 10.1074/jbc.M800102200 PMC2447655

[17] White E , Mehnert JM , Chan CS . Autophagy, metabolism, and cancer. Clin Cancer Res. 2015;21:5037‐5046. 26567363 10.1158/1078-0432.CCR-15-0490 PMC4646728

[18] Zhao JF , Zhao Q , Hu H , et al. The ASH1‐miR‐375‐YWHAZ signaling axis regulates tumor properties in hepatocellular carcinoma. Mol Ther Nucleic Acids. 2018;11:538‐553. 29858089 10.1016/j.omtn.2018.04.007 PMC5944419

[19] Weerasekara VK , Panek DJ , Broadbent DG , et al. Metabolic‐stress‐induced rearrangement of the 14‐3‐3zeta interactome promotes autophagy via a ULK1‐ and AMPK‐regulated 14‐3‐3zeta interaction with phosphorylated Atg9. Mol Cell Biol. 2014;34:4379‐4388. 25266655 10.1128/MCB.00740-14 PMC4248729

[20] Yang X , Lee WH , Sobott F , et al. Structural basis for protein‐protein interactions in the 14‐3‐3 protein family. Proc Natl Acad Sci USA. 2006;103:17237‐17242. 17085597 10.1073/pnas.0605779103 PMC1859916

[21] Yaffe MB , Rittinger K , Volinia S , et al. The structural basis for 14‐3‐3:phosphopeptide binding specificity. Cell. 1997;91:961‐971. 9428519 10.1016/s0092-8674(00)80487-0

[22] Song X , Lee DH , Dilly AK , et al. Crosstalk between apoptosis and autophagy is regulated by the Arginylated BiP/Beclin‐1/p62 complex. Mol Cancer Res. 2018;16:1077‐1091. 29669822 10.1158/1541-7786.MCR-17-0685 PMC6030503

[23] Wang T , Hu HS , Feng YX , et al. Characterisation of a novel cell line (CSQT‐2) with high metastatic activity derived from portal vein tumour thrombus of hepatocellular carcinoma. Br J Cancer. 2010;102:1618‐1626. 20461085 10.1038/sj.bjc.6605689 PMC2883151

[24] Yang J , Qin LX , Li Y , et al. Molecular cytogenetic characteristics of the human hepatocellular carcinoma cell line HCCLM3 with high metastatic potential: comparative genomic hybridization and multiplex fluorescence in situ hybridization. Cancer Genet Cytogenet. 2005;158:180‐183. 15796966 10.1016/j.cancergencyto.2004.05.010

[25] Zalckvar E , Berissi H , Mizrachy L , et al. DAP‐kinase‐mediated phosphorylation on the BH3 domain of beclin 1 promotes dissociation of beclin 1 from Bcl‐XL and induction of autophagy. EMBO Rep. 2009;10:285‐292. 19180116 10.1038/embor.2008.246 PMC2658558

[26] Fogel AI , Dlouhy BJ , Wang C , et al. Role of membrane association and Atg14‐dependent phosphorylation in beclin‐1‐mediated autophagy. Mol Cell Biol. 2013;33:3675‐3688. 23878393 10.1128/MCB.00079-13 PMC3753860

[27] Qian X , Li X , Cai Q , et al. Phosphoglycerate kinase 1 phosphorylates beclin1 to induce autophagy. Mol Cell. 2017; 65:917‐931.e6. 28238651 10.1016/j.molcel.2017.01.027 PMC5389741

[28] Barbash O , Lee EK , Diehl JA . Phosphorylation‐dependent regulation of SCF(Fbx4) dimerization and activity involves a novel component, 14‐3‐3varepsilon. Oncogene. 2011;30:1995‐2002. 21242966 10.1038/onc.2010.584 PMC3084329

[29] Wang B , Liu K , Lin FT , Lin WC . A role for 14‐3‐3 tau in E2F1 stabilization and DNA damage‐induced apoptosis. J Biol Chem. 2004;279:54140‐54152. 15494392 10.1074/jbc.M410493200 PMC3904440

[30] Xue D , Yang Y , Liu Y , et al. MicroRNA‐206 attenuates the growth and angiogenesis in non‐small cell lung cancer cells by blocking the 14‐3‐3zeta/STAT3/HIF‐1alpha/VEGF signaling. Oncotarget. 2016;7:79805‐79813. 27806334 10.18632/oncotarget.12972 PMC5346752

[31] Mohammad DK , Nore BF , Hussain A , Gustafsson MO , Mohamed AJ , Smith CI . Dual phosphorylation of Btk by Akt/protein kinase b provides docking for 14‐3‐3zeta, regulates shuttling, and attenuates both tonic and induced signaling in B cells. Mol Cell Biol. 2013;33:3214‐3226. 23754751 10.1128/MCB.00247-13 PMC3753922

[32] Volinia S , Dhand R , Vanhaesebroeck B , et al. A human phosphatidylinositol 3‐kinase complex related to the yeast Vps34p‐Vps15p protein sorting system. EMBO J. 1995;14:3339‐3348. 7628435 10.1002/j.1460-2075.1995.tb07340.x PMC394401

[33] Itakura E , Kishi C , Inoue K , Mizushima N . Beclin 1 forms two distinct phosphatidylinositol 3‐kinase complexes with mammalian Atg14 and UVRAG. Mol Biol Cell. 2008;19:5360‐5372. 18843052 10.1091/mbc.E08-01-0080 PMC2592660

[34] Russell RC , Tian Y , Yuan H , et al. ULK1 induces autophagy by phosphorylating Beclin‐1 and activating VPS34 lipid kinase. Nat Cell Biol. 2013;15:741‐750. 23685627 10.1038/ncb2757 PMC3885611

[35] Wang S , Li J , Du Y , et al. The Class I PI3K inhibitor S14161 induces autophagy in malignant blood cells by modulating the Beclin 1/Vps34 complex. J Pharmacol Sci. 2017;134:197‐202. 28779993 10.1016/j.jphs.2017.07.001

[36] Kumar A , Singh UK , Chaudhary A . Targeting autophagy to overcome drug resistance in cancer therapy. Future Med Chem. 2015;7:1535‐1542. 26334206 10.4155/fmc.15.88

[37] Fan T , Li R , Todd NW , et al. Up‐regulation of 14‐3‐3zeta in lung cancer and its implication as prognostic and therapeutic target. Cancer Res. 2007;67:7901‐7906. 17699796 10.1158/0008-5472.CAN-07-0090

[38] Bergamaschi A , Katzenellenbogen BS . Tamoxifen downregulation of miR‐451 increases 14‐3‐3zeta and promotes breast cancer cell survival and endocrine resistance. Oncogene. 2012;31:39‐47. 21666713 10.1038/onc.2011.223 PMC3175015

[39] Choi JE , Hur W , Jung CK , et al. Silencing of 14‐3‐3zeta over‐expression in hepatocellular carcinoma inhibits tumor growth and enhances chemosensitivity to cis‐diammined dichloridoplatium. Cancer Lett. 2011;303:99‐107. 21334806 10.1016/j.canlet.2011.01.015

