# Supplementary figures and images for: 14‐3‐3ζ binds to and stabilizes phospho‐beclin 1S295 and induces autophagy in hepatocellular carcinoma cells
Source: J Cell Mol Med. 2019 Nov 11;24(1):954–64. doi: 10.1111/jcmm.14806 (PMC6933394; doi:10.1111/jcmm.14806)

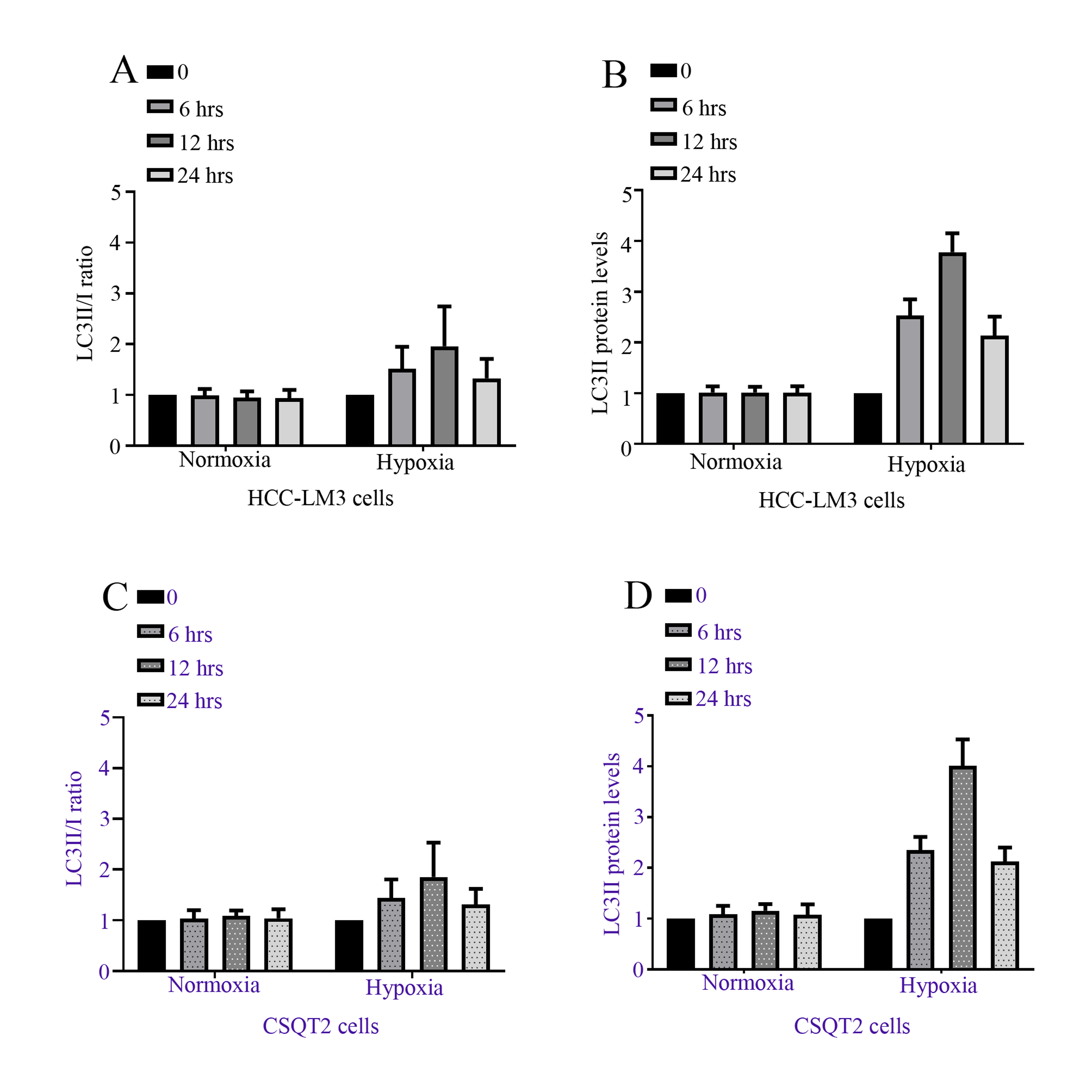

Supplement: Supplementary file 1 [file JCMM-24-954-s001.tif]

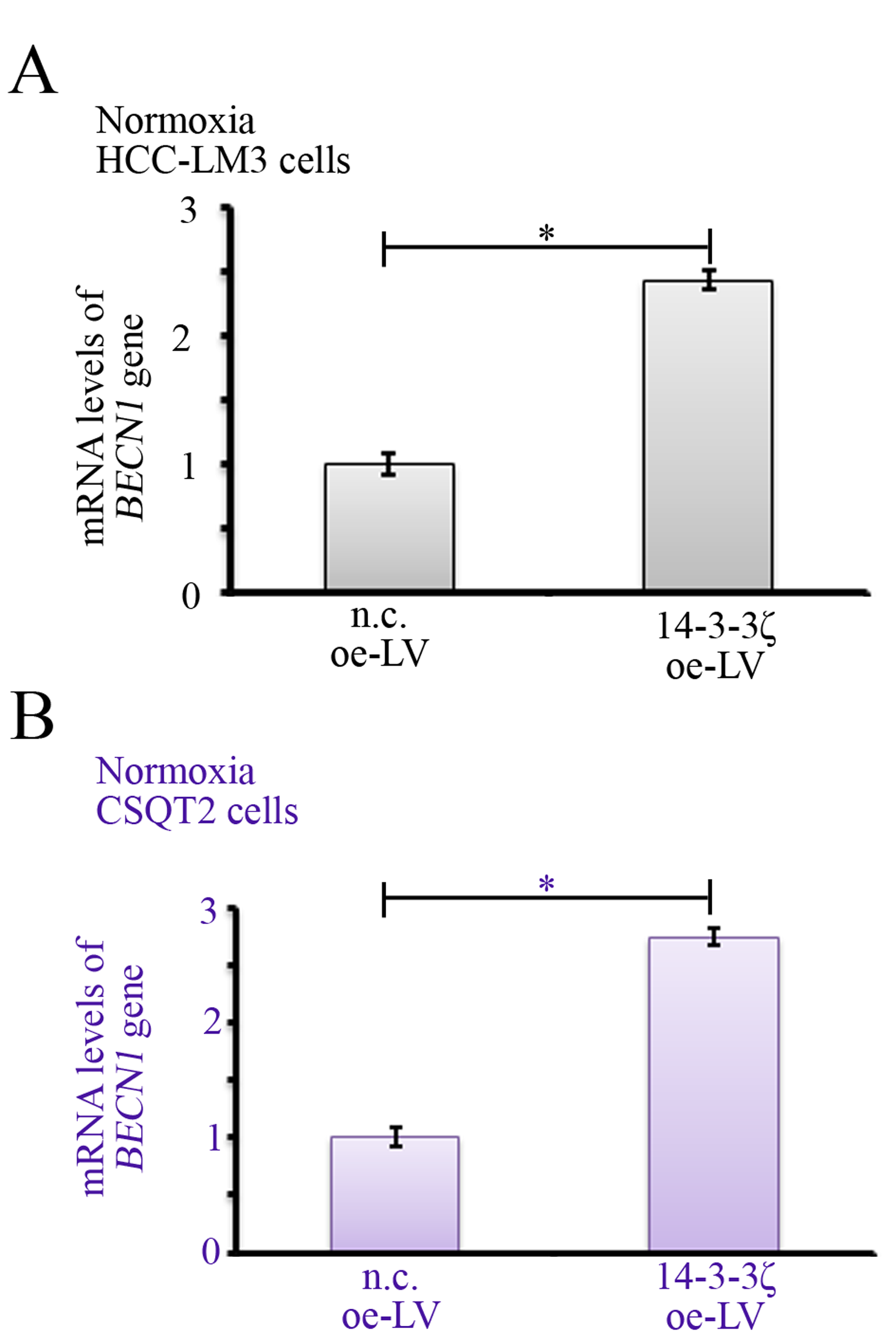

Supplement: Supplementary file 2 [file JCMM-24-954-s002.tif]

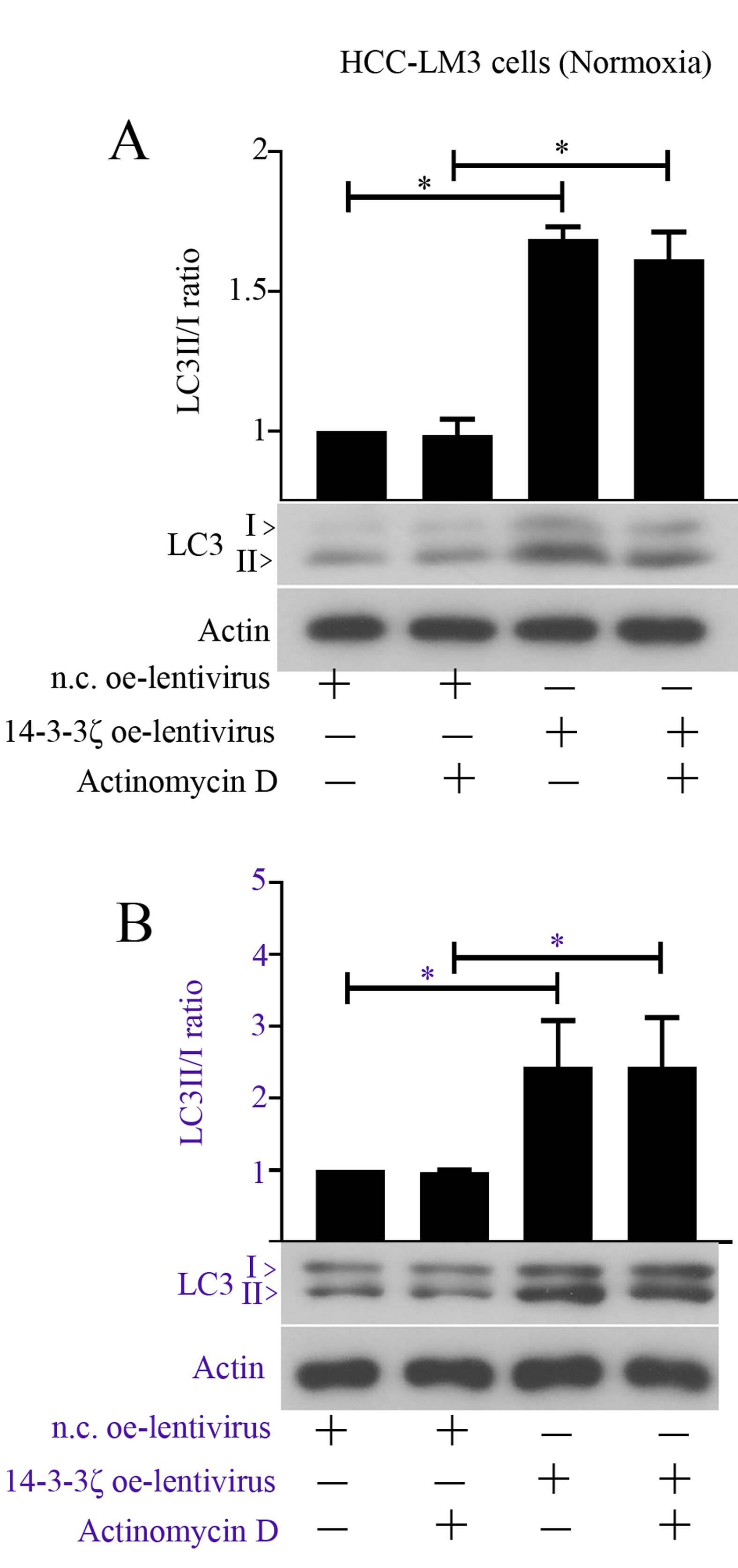

Supplement: Supplementary file 3 [file JCMM-24-954-s003.tif]
